# QTL Mapping and Candidate Gene Analysis for Alkali Tolerance in *Japonica* Rice at the bud Stage Based on Linkage Mapping and Genome-Wide Association Study

**DOI:** 10.1186/s12284-020-00412-5

**Published:** 2020-07-16

**Authors:** Xianwei Li, Hongliang Zheng, Wenshen Wu, Hualong Liu, Jingguo Wang, Yan Jia, Jiaming Li, Luomiao Yang, Lei Lei, Detang Zou, Hongwei Zhao

**Affiliations:** Key Laboratory of Germplasm Enhancement, Physiology and Ecology of Food Crops in Cold Region, Ministry of Education, Northeast Agricultural University, Harbin, 150030 China

**Keywords:** *Japonica* rice, The bud stage, Alkalinity tolerance, Linkage mapping, Genome-wide association study

## Abstract

**Background:**

Salinity-alkalinity stress is one of the major factors limiting rice production. Damage caused by alkaline salt stress is more severe than that caused by neutral salt stress. Alkali tolerance at the bud stage in rice directly affects seedling survival and final yield when using the direct seeding cultivation model. However, genetic resources (QTLs and genes) for rice breeders to improve alkali tolerance are limited. In this study, we combined linkage mapping and a genome-wide association study (GWAS) to analyze the genetic structure of this trait in *japonica* rice at the bud stage.

**Results:**

A population of 184 recombinant inbred lines (RILs) was utilized to map quantitative trait loci (QTLs) for the root length under control condition (RL), alkaline stress (ARL) and relative root length (RRL) at the bud stage. A major QTL related to alkali tolerance at the rice bud stage, *qAT11*, was detected on chromosome 11. Interestingly, a GWAS identified a lead SNP (Chr_21,999,659) in *qAT11* that was significantly associated with alkaline tolerance. After filtering by linkage disequilibrium (LD), haplotype analysis, quantitative real-time PCR, we obtained three candidate genes (*LOC_Os11g37300*, *LOC_Os11g37320* and *LOC_Os11g37390*). In addition, we performed phenotype verification on the CRISPR/Cas9 mutant of *LOC_Os11g37390*.

**Conclusion:**

Based on these results, *LOC_Os11g37300*, *LOC_Os11g37320* and *LOC_Os11g37390* were the candidate genes contributing to alkaline tolerance in *japonica* rice. This study provides resources for breeding aimed at improving rice responses to alkalinity stress.

## Background

Soil salinity-alkalinity is one of the most important abiotic stresses limiting agricultural production. About 80 million hectares of irrigated soil worldwide are damaged by saline and alkaline stress, accounting for about 40% of the total irrigated area. With the increasing human population, industrial modernization, and deterioration of the ecological environment, areas of reclamation are expanding and areas of soil salinity-alkalinity are increasing. It is estimated that by 2050, soil salinization will destroy 50% of agricultural land in the world (Qadir et al. 2014). Rice (*Oryza sativa* L.) is one of the most important staple crops, feeding more than 60% of the population in China and providing 20% of energy per capita on a global scale (Cheng et al., 2007). It is also a salt-alkali-sensitive crop. Soil salinity-alkalinity can negatively affect rice seed germination, growth, seed maturation, the seed setting rate, and ultimately the rice yield (Zhu 2001; Shi et al. 2017). Therefore, studying salinity-alkalinity tolerance in rice has important practical significance for breeding tolerant cultivars and improving the utilization of saline-alkaline land (Li et al. 2014).

Soluble salts in saline-alkali soil mainly include cations, such as Na^+^, Ca^2+^, Mg^2+^, and K^+^, and anions, such as CO_3_^2−^, HCO_3_^−^, Cl^−^, SO_4_^2−^, and NO_3_^−^, all of which come from neutral or alkaline salts. Stress caused by alkaline salts, such as Na_2_CO_3_ and NaHCO_3_, as well as high pH stress affect plants. Damage by alkaline salts is significantly greater than that caused by neutral salts, such as NaCl and Na_2_SO_4_, with distinct mechanisms of action (Guo et al. 2014). Recent research has increasingly focused on the serious environmental problem of alkaline stress. Soda saline-alkaline soil grassland in Northeast China has reached 70% of the total area and continues to expand. Therefore, the development of rice production on saline-alkali land has great significance for improving saline-alkali land and food security.

Owing to the low labor requirements and high efficiency, the direct seeding method has become an important cultivation model. Rice alkali tolerance at the bud stage is a major determinant of growth stability in saline-alkaline soil using the direct seeding cultivation model. During the planting season, rice seeds sensitive to alkali conditions at the bud stage exhibit low seedling rates and even seed death. Thus, improving the alkaline tolerance of rice at the bud stage is an important objective in rice breeding.

Salinity-alkalinity tolerance is a quantitative genetic characteristic that is controlled by multiple genes in rice (Qi et al. 2008). Extensive research has focused on the mechanism under lying salt tolerance in rice (Wang et al. 2011; Wang et al. 2012a; Sun et al. 2014). These studies have evaluated growth, morphology, physiology, and biochemistry, and some salt-tolerant genes have been identified by map-based cloning, such as *SKC1* (Ren et al. 2005) and *DST* (Huang et al. 2009). However, less progress has been made in the localization of QTLs for alkali stress (NaHCO_3_ or Na_2_CO_3_), and most of this research is in the stage of primary QTL mapping. For example, using 200 F_2: 3_ individuals, 13 and 6 QTLs related to the dead leaf rate and dead seedling rate under alkaline conditions, respectively, have been identified (Qi et al. 2008). A common QTL associated with the score of alkalinity tolerance and shoot Na^+^ and K^+^ concentrations in the rice seedling stage explained 13.36–13.64% of phenotypic variation and was identified as *OsIRO3* (Li et al. 2019). Cheng et al. (2008) detected 14 QTLs in 0.15% Na_2_CO_3_ alkaline solution at the germination and early seedling stages. A previous study detected seven QTLs under alkaline stress, among which *qSNC3* explained 21.24% of the total phenotypic (Li et al. 2017). Additional genetic resources (QTLs/genes) that can be used by rice breeders to improve alkalinity tolerance are needed.

Bi-parental QTL mapping and GWAS are effective and precise tools for the detection of QTLs for complex traits (Wang et al. 2011; Kumar et al. 2015; Liang et al. 2015; Zheng et al. 2015; Shakiba et al. 2017; Shi et al. 2017). Combining the two methods can improve the breadth and accuracy of QTL detection. The combination of association analyses and linkage mapping for gene mining has been highly successful for studies of quantitative traits in rice. For example, linkage mapping and an association analysis have been used to detect a major QTL (*LP1*) controlling panicle length on chromosome 9, which was further narrowed to a 90-kb region using a NIL-F2 population and analyzed by sequencing (Liu et al. 2016). A similar strategy was used to analyze the genetic structure of rice tolerance to aluminum toxicity, resulting in the identification of two QTLs (Famoso et al. 2011). These studies demonstrate the feasibility of identifying QTL/genes for alkali tolerance by combined linkage mapping and GWAS.

In this study, the root length under control condition (RL), alkaline stress (ARL) and relative root length (RRL) were used to assess the genetic basis of alkali tolerance in rice by linkage mapping and GWAS at the bud stage. A major QTL, *qAT11*, on chromosome 11 was detected by linkage mapping, and Chr11_21,999,659 within *qAT11* was significantly associated with alkali tolerance, as determined by a GWAS. According to an LD analysis of the whole genome, a 218-kb region on chromosome 11 was selected for further analysis. Finally, we used haplotype analysis and gene expression analysis to obtained three candidate genes (*LOC_Os11g37300*, *LOC_Os11g37320* and *LOC_Os11g37390*) which were most likely involved in the regulation of alkali tolerance in rice.

## Results

### Phenotypic Variation

**Fig. 1:**
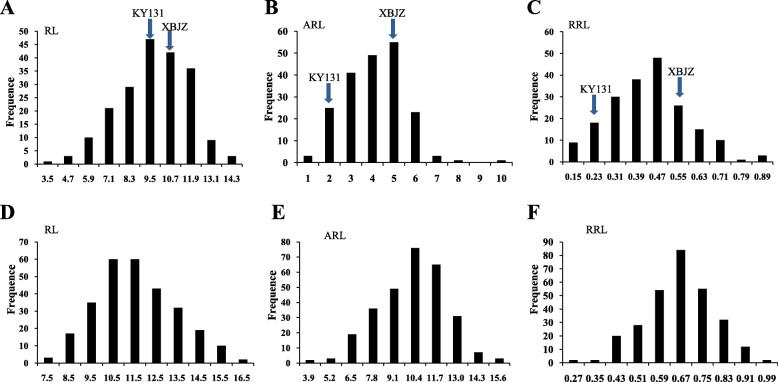
Phenotypic variation in the RL, ARL, and RRL in RILs and 295 *japonica* rice varieties. **a**, **b** and **c** represent the RL, ARL, and RRL of RILs, respectively; **d**, **e**, and **f** represent the RL, ARL, and RRL of natural populations, respectively

The means, standard deviations, and range of root lengths (RL), ARL, and RRL at the bud stage of RIL and natural populations are presented in Table S1. The mean ARLs were lower than RLs, indicating that alkali stress depressed the growth and development of *japonica* rice at the bud stage. Two parents of the RILs, Kongyu131 (KY131) and Xiaobaijingzi (XBJZ), showed different tolerances to alkali stress (Table S1). The ARL and RRL of KY131 were lower than those of XBJZ, indicating that XBJZ was more tolerant to alkali stress than KY131 at the bud stage. The mean RRL varied from 0.11 to 0.89 in the RIL population and from 0.25 to 0.94 in the natural population. The phenotypic values of RL, ARL and RRL in the two populations approximately followed a normal distribution, indicating that these indices are quantitative traits controlled by multiple factors (Fig. 1).

### Linkage Mapping for Alkali Tolerance at the Bud Stage

In total, 527 markers were used for QTL mapping of RL, ARL and RRL. Eight QTLs associated with the RL, ARL and RRL were detected on chromosomes 1, 4, 6, and 11 (Table 1 and Fig. S1), with LOD values of 2.76 to 7.55 and proportions of phenotypic variance explained from 4.03% to 18.75%. *qARL6* and *qRRL6–2* were located in the same interval and were considered the same QTL (named *qAT6*). *qAT6* was located in the physical region between markers C6_10554837 and C6_19920701 and explained 6.35%–6.87% of the phenotypic variation. Similarly, *qARL11* and *qRRL11* were considered the same QTL (named *qAT11*). *qAT11* was located between markers C11_21685006 and C11_22560140 and explained 11.38%–18.75% of the phenotypic variation.

**Table 1:** QTLs for RL, ARL and RRL based on the mean trait values of three replications in 184 RILs

Traits	QTLs	Left Marker	Right Marker	Chr.	LOD	*R*^*2*^(%)	Additive effect	QTL in previous study
RL	*qRL1*	C1_4655517	C1_4984609	1	2.76	4.03	0.41	
	*qRL4*	C4_32090432	C4_32195798	4	3.30	7.75	−0.56	
ARL	*qARL4*	C4_32090432	C4_32195798	4	4.32	7.73	−0.39	*qIR4* (Wang et al. 2011) *qPH4* (Thomson et al. 2010)
	*qARL6*	C6_10554837	C6_19920701	6	3.52	6.87	0.37	*qDRW6* (Wang et al. 2012b).
	*qARL11*	C11_21685006	C11_22560140	11	6.03	11.38	−0.48	*qSKC11* (Zang et al. 2008)
RRL	*qRRL6–1*	C6_4815311	C6_4915271	6	3.15	4.05	−3.03	
	*qRRL6–2*	C6_10554837	C6_19920701	6	3.96	6.35	3.72	*qDRW6* (Wang et al. 2012b).
	*qRRL11*	C11_21685006	C11_22560140	11	7.55	18.75	−6.45	*qSKC11* (Zang et al. 2008)

R^2^ (%): Phenotypic variance explained

### GWAS for Alkali Tolerance-Related Traits in a Natural Population

**Fig. 2:**
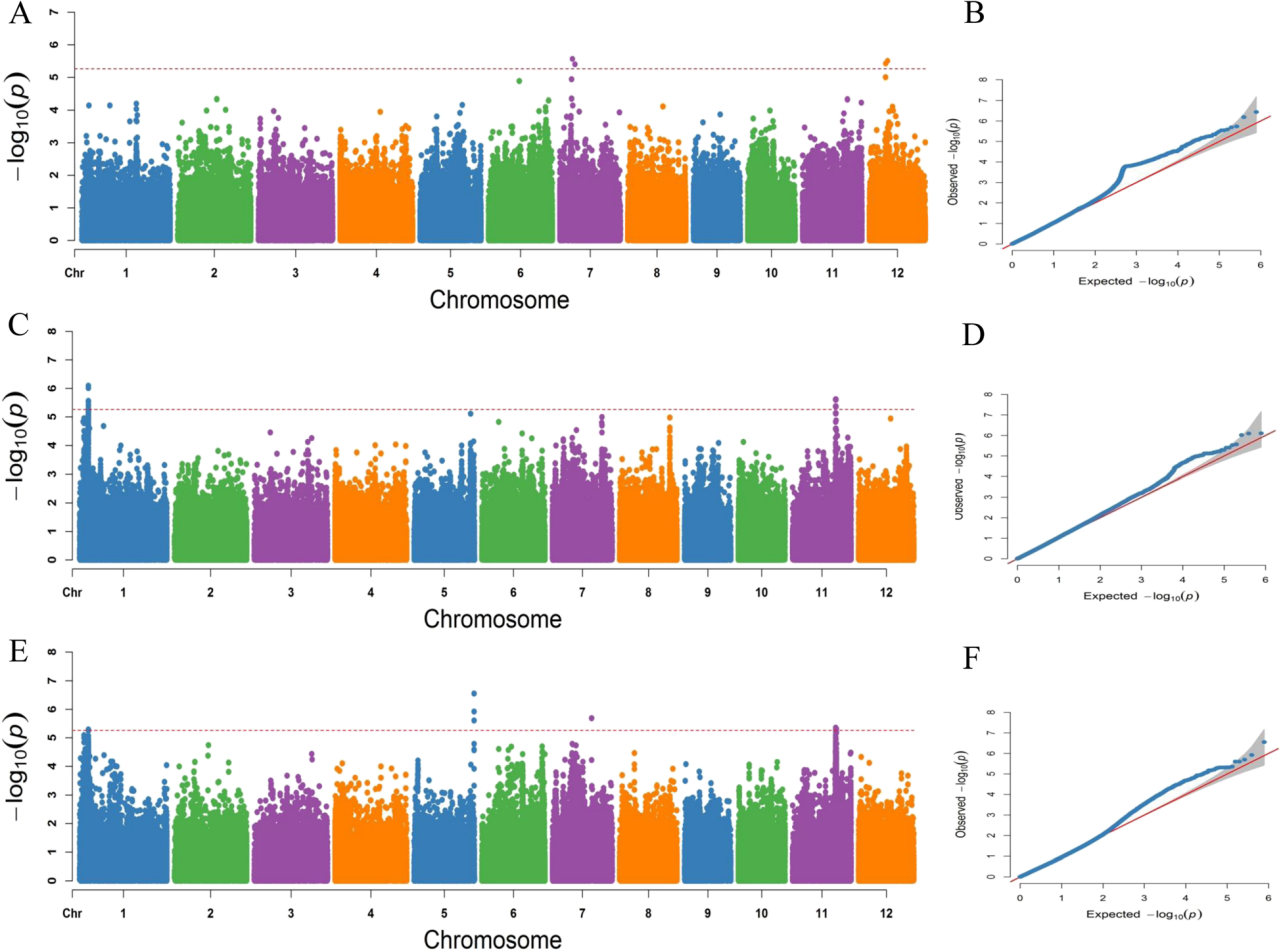
Manhattan plots and quantile–quantile (Q-Q) plots of GWAS for the RL, ARL and RRL. **a**, Manhattan plot for the RL. **b**, Q-Q plot for the RL. **c,** Manhattan plot for the ARL. **d**, Q-Q plot for ARL. **e**, Manhattan plot for the RRL. **f**, Q-Q plot for RRL

**Fig. 3:**
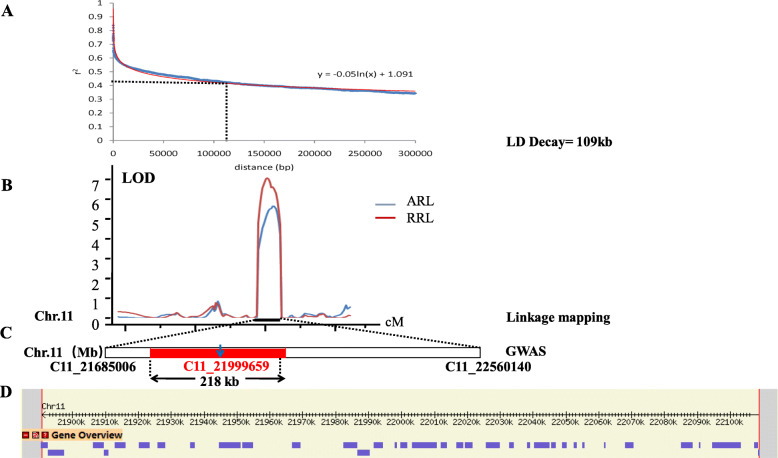
Identification of candidate genes by linkage mapping and GWAS. **a** LD decay analysis of the whole genome in 295 *japonica* rice varieties. When r^2^ decays to half of the maximum value, the corresponding physical distance (109 kb) is recorded as the LD attenuation distance of the whole genome. **b** A QTL related to alkali tolerance on chromosome 11 was identified in 184 RILs and mapped to the interval between the markers C11_21685006 and C11_22560140 by linkage mapping. **c** Physical location of the lead SNP (C11_21,999,659) on chromosome 11 detected by the GWAS. **d** 35 genes in the 218 kb region

A GWAS was conducted with 788,369 SNPs obtained from previous studies in our laboratory (Li et al. 2019). Manhattan and quantile–quantile plots for the GWAS results are shown in Fig. 2. Nine lead SNPs significantly associated with RL, ARL and RRL are listed in Table 2. These SNPs were located on chromosomes 1, 5, 7, 11 and 12 with *R*^*2*^ values ranging from 6.64% to 10.20%. One lead SNP, Chr1_4,321,954, was located on chromosome 1 and was associated with ARL and RRL (*R*^*2*^ = 8.82–10.20%). The lead SNP, Chr11_21,999,659, associated with both ARL and RRL on chromosome 11 was located in *qAT11* identified by the previous linkage mapping (Fig. 3b and c). According to a genome-wide LD decay analysis (Fig. 3a), we obtained a 218-kb region that was overlapping in linkage mapping and the GWAS. This interval is critical for alkali tolerance at the bud stage of rice and likely contains a candidate gene for this trait.

**Table 2:** Lead SNPs for RL, ARL and RRL identified by GWAS

Traits	Lead SNP	Chromosome	Position	*P* value	*R*^*2*^(%)	QTL in previous study
RL	Chr7__6,215,545	7	6,215,545	5.07E-06	6.64	
	Chr12__7,813,381	12	7,813,381	5.16E-06	7.97	
ARL	Chr1__4,321,954	1	4,321,954	8.00E-07	10.20	*qRL1* (Sabouri and Sabouri 2008)
	Chr11__21,999,659	11	21,999,659	9.74E-07	10.05	*qSKC11* (Zang et al. 2008)
RRL	Chr1__4,321,954	1	4,321,954	5.11E-06	8.82	*qRL1* (Sabouri and Sabouri 2008)
	Chr5__29,730,673	5	29,730,673	2.84E-07	9.59	*qRL5* (Sabouri and Sabouri 2008)
	Chr7__19,513,614	7	19,513,614	2.04E-06	8.15	*qRL7* (Sabouri and Sabouri 2008)
	Chr11__21,381,167	11	21,381,167	4.43E-06	9.42	
	Chr11__21,999,659	11	21,999,659	4.84E-06	10.19	*qSKC11* (Zang et al. 2008)

R^2^ (%): Phenotypic variance explained

### Haplotype Analysis of Candidate Genes

**Fig. 4:**
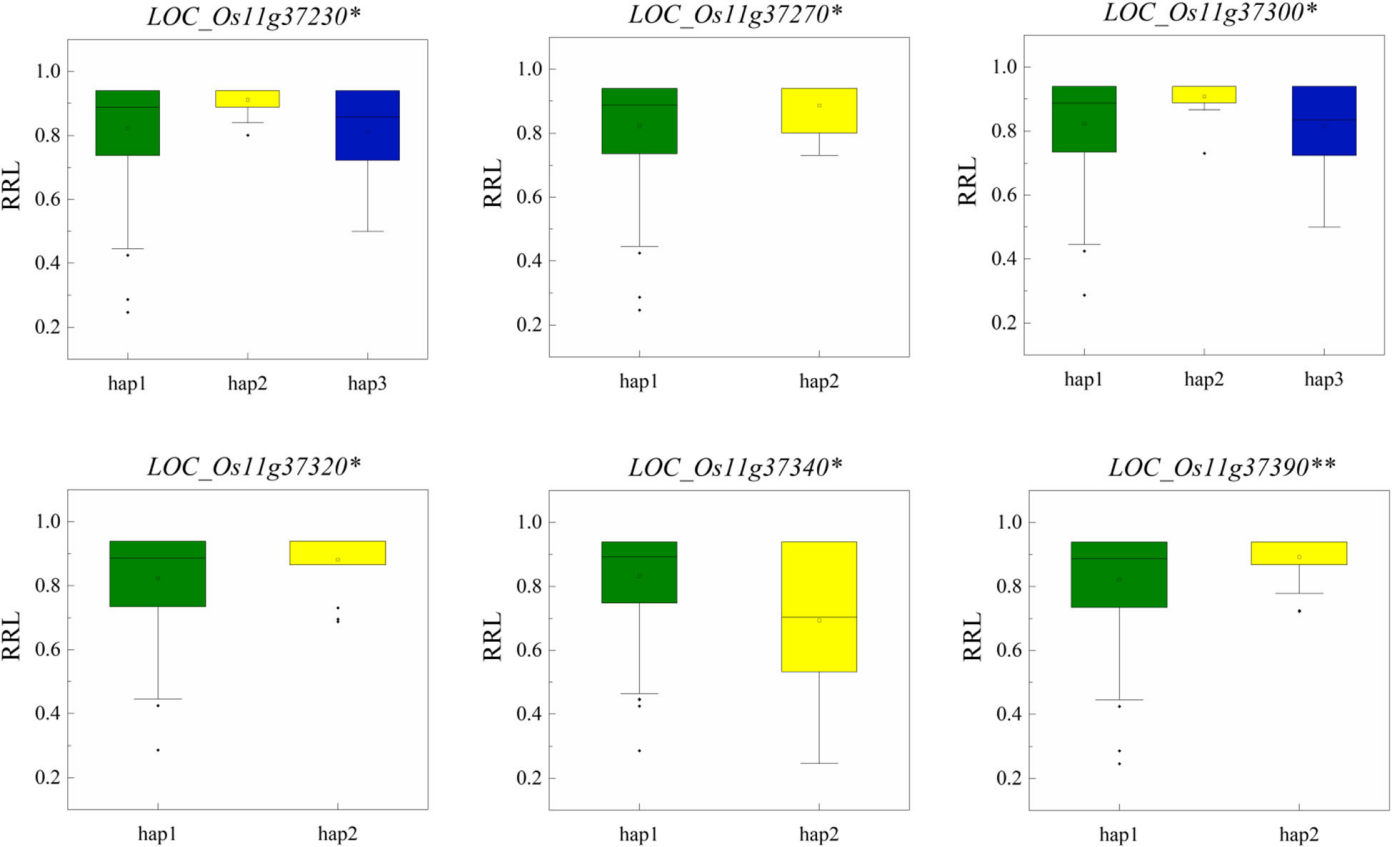
Boxplots for RRL based on the haplotypes (Hap) for candidate genes; green, yellow and blue indicate the phenotypic result for hap1, hap2 and hap3, respectively (The * and ** suggest significance of ANOVA at *P* < 0.05 and *P* < 0.01, respectively)

The 218-kb region contained 35 genes, including 17 functionally annotated genes, 9 genes encoding proteins with unknown functions, 2 genes encoding hypothetical proteins, and 7 genes encoding retrotransposons (Fig. 3d and Table S2). We conducted haplotype analysis on non-synonymous mutant SNPs of the exon region and SNPs of the promoter to identify important candidate genes in the above interval. Six genes (*LOC_Os11g37230*, *LOC_Os11g37270*, *LOC_Os11g37300*, *LOC_Os11g37320*, *LOC_Os11g37340* and *LOC_Os11g37390*) were associated with significant differences in the RRL among different haplotypes (Fig. 4). Of the six genes, only *LOC_Os11g37320* was divided into two haplotypes by SNP in the promoter region, and the other genes were divided into two or three haplotypes by non-synonymous mutation SNPs in the exon region.

Three haplotypes were found for *LOC_Os11g37230* and *LOC_Os11g37300.* Haplotypes analysis revealed that significant difference for RRL was observed between hap1 (TT) and hap2 (GT) in *LOC_Os11g37230* (Table 3 and Fig. 4). Similarly, significant difference existed between hap1 (CTC) and hap2 (TCT) in *LOC_Os11g37300* (Table 3 and Fig. 4). Two haplotypes were found for *LOC_Os11g37270*, *LOC_Os11g37320*, *LOC_Os11g37340* and *LOC_Os11g37390*, significant differences for RRL between different haplotypes were observed for these four genes (Table 3 and Fig. 4).

**Table 3:** Candidate gene haplotype group and the composition of each haplotype SNP

Gene	hap1/ Number	hap2/ Number	hap3/ Number
*LOC_Os11g37230*	TT/261(XBJZ)	GT/10(KY131)	GA/10
*LOC_Os11g37270*	A/264(KY131)	G/14(XBJZ)	
*LOC_Os11g37300*	CTC/250(KY131)	TCT/13(XBJZ)	CCC/12
*LOC_Os11g37320*	C/271(KY131)	T/15(XBJZ)	
*LOC_Os11g37340*	CT/272(XBJZ)	TC/15(KY131)	
*LOC_Os11g37390*	A/269(KY131)	T/17(XBJZ)	

### Identification of Candidate Genes by Gene Expression and Sequence Analysis

**Fig. 5:**
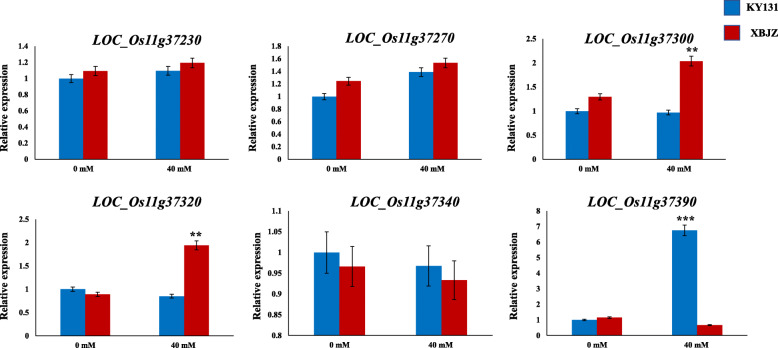
Expression patterns of six genes under normal growth conditions and alkali stress (**P < 0.01; ***P < 0.001; Student’s *t*-test)

According to the results of haplotype analysis, we evaluated the six genes by qRT-PCR in KY131 and XBJZ under alkali stress and control conditions (Fig. 5), and performed sequence analysis. Under normal condition, there was no significant difference in the expression levels of the six genes between KY131 and XBJZ. However, under alkalinity stress, three genes (*LOC_Os11g37300*, *LOC_Os11g37320*, and *LOC_Os11g37390*) were differentially expressed between KY131 and XBJZ (Fig. 5). *LOC_Os11g37390* showed higher expression levels in KY131 than in XBJZ. The opposite expression pattern was observed for *LOC_Os11g37300* and *LOC_Os11g37320* (Fig. 5). In fact, the expression level of *LOC_Os11g37390* in KY131 was nearly 10-fold higher than that in XBJZ after treatment with 40 mM NaHCO_3_, whereas only a 1–2-fold change was found for the other two genes. Of these three genes, *LOC_Os11g37300* and *LOC_Os11g37390* belong to the F-box gene family, which is influenced by salt stress (Jain et al. 2007).

We further sequenced the six genes (including gene and promoter region) in KY131 and XBJZ. *LOC_Os11g37390* of KY131 contained 1-bp insertion of a G base in the CDS region compared with the sequence in XBJZ. Other five genes exhibited no sequence differences between KY131 and XBJZ.

### *LOC_Os11g37390* Mutant Is Sensitive to Alkali Stress

**Fig. 6:**
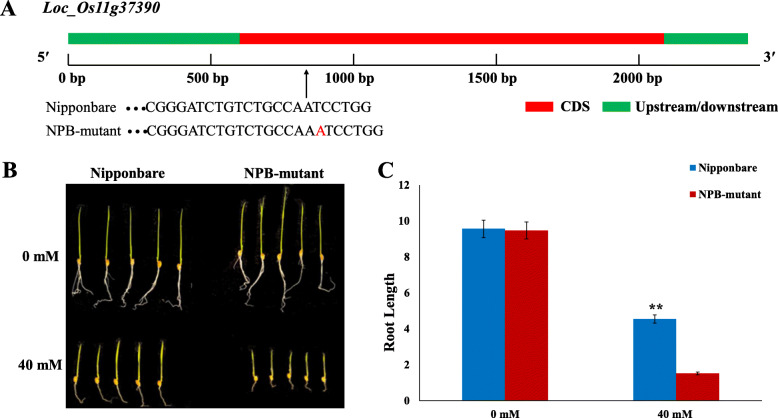
**a** DNA sequence of *LOC_Os11g37390* in Nipponbare and NPB-mutant. **b** Growth of NPB-mutant and control shoots treated with 0 or 40 mM NaHCO_3_ for 10 days. **c** Significant differences in phenotypes between wild-type and NPB-mutant (**P < 0.01; Student’s *t*-test)

To confirm the function of *LOC_Os11g37390* in response to alkali stress, mutant named NPB-mutant, was obtained. The A base was inserted at 813 bp of the CDS region (Fig. 6a). The growth of NPB-mutant (Nipponbare background) was similar to that of Nipponbare; however, NPB-mutant was hypersensitive to alkali stress by 40 mM NaHCO_3_ treatment (Fig. 6b).

In the presence of 0 mM NaHCO_3_, no apparent difference was found between the NPB-mutant and Nipponbare plants roots, indicating that *LOC_Os11g37390* (*OsFBDUF54*) had negligible effect on the growth of rice roots under normal conditions (Fig. 6b). However, after exposure to 40 mM NaHCO_3_ for 10 days, obvious differences were observed between the NPB-mutant and wild-type plants. The NPB-mutant plants were more sensitive to alkali stress than the wild-type plants. Under alkaline stress, the root lengths of the NPB-mutant plants were significantly shorter than those of the wild-type plants (Fig. 6b and c). Thus, *LOC_Os11g37390* is the most likely candidate gene involved in the regulation of alkali tolerance in rice.

## Discussion

Land and water productivity are seriously affected by saline and alkali stress, which obviously reduce crop production, such as soybean (Do et al. 2016), wheat (Francois et al. 1986), and rice (Liang et al. 2017). In contrast to salinity, alkalinity not only causes ionic toxicity in plants but also damages the stability of cells due to its high pH, thus affecting the normal growth of plants (Chen et al. 2009). Many methods have been used to assess alkalinity tolerance in rice, and root length is a common phenotypic indicator (Liang et al. 2015). Plant roots are the main organs by which plants absorb water and nutrients. They have various biological functions, such as fixing above-ground parts, decomposing toxic substances, and sensing environmental stimuli or stress (Kreszies et al. 2018). Analyses of root length at the bud stage in rice under alkaline stress provide a basis for breeding in saline-alkali land from the perspective of the root system, addressing the lack of rice germplasm resources for saline-alkali land.

Salinity-alkalinity tolerance is a highly complex trait in rice (Zhang et al. 2013). Many QTLs related to salt tolerance have been identified. However, few QTLs related to tolerance to alkali stress have been reported. Many different traits have been used as physical indexes for mapping QTLs for salt tolerance, and previously mapped loci overlapped with or were in close proximity to the loci identified in our study based on physical distance. A previous research detected three QTLs (*qRL1*, *qRL5*, and *qRL7*) on chromosomes 1, 5, and 7; the lead SNPs chr1_4,321,954, chr5_ 29,730,673, and chr7_ 19,513,614 detected by our GWAS were located within or close to *qRL1*, *qRL5*, and *qRL7* (Sabouri and Sabouri 2008). It was found that *qIR4* on chromosome 4 accounts for 22.9% of the phenotypic variation in the imbibition rate under salt stress and *qPH4* flanked by RM17391 and RM127 explains 25% of the phenotypic variation for seedling height (Thomson et al. 2010; Wang et al. 2011). These two QTLs harbored *qARL4* in our study by linkage mapping. *qDRW6*, associated with dry root weight on chromosome 6, contained *qAT6*, detected in our study by linkage mapping (Wang et al. 2012b). Another QTL, *qSKC11* (Zang et al. 2008), contained *qAT11* and Chr11__21,999,659, identified in our study identified by linkage mapping and GWAS. These results supported the accuracy of our results.

In particular, we identified *qAT11* and Chr11__21,999,659 by linkage mapping and GWAS on chromosome 11. According to the genome-wide LD decay of the GWAS (Fig. 3a), we identified a region of approximately 218 kb as a candidate region for further studies and this was the overlapping part in linkage mapping and the GWAS (Li et al. 2019). Based on haplotype analysis, six genes were associated with significant differences in the RRL among different haplotypes, on the basis of gene expression analysis, we obtained three candidate genes (*LOC_Os11g37300* named *OsFBDUF53*, *LOC_Os11g37320* named *LTPL79*, and *LOC_Os11g37390* named *OsFBDUF54*). Of the three candidate genes, *LTPL79* is a protease inhibitor gene, and its function has not been preliminary studied, *OsFBDUF53* and *OsFBDUF54* belong to the F-box gene family, which is influenced by salt stress (Jain et al. 2007). Through sequence analysis of candidate genes we found that *OsFBDUF54* of KY131 contained 1-bp insertion of a G base in the CDS region compared with the sequence in XBJZ. However, the other two genes (*OsFBDUF53* and *LTPL79*) with different gene expression levels, even if there is no difference in nucleotide sequence, it can be a causal gene if the gene expression level is different. Through further integrated analysis, we selected *OsFBDUF54* to conducted the preliminary phenotype verification of NPB-mutants, we identified a most likely candidate gene for alkalinity tolerance in rice.

F-box proteins, characterized by a conserved F-box motif of approximately 40 amino acids, represent one of the largest protein families, with about 700 members in *Arabidopsis* and rice (Gagne et al. 2002; Jain et al. 2007). In plants, only a small portion of F-box proteins have been studied; they have important roles in the regulation of various developmental processes and stress responses by integrating almost all phytohormone signaling pathways (Lechner et al. 2006; Dreher and Callis 2007; Zhang et al. 2008). Therefore, it is possible to speculate that they play important roles in a series of stress responses throughout the plant life cycle.

In the present study, the RRL of KY131 and XBJZ were 0.18 and 0.48, respectively, and there were significant differences between the different haplotypes of *OsFBDUF53*, *LTPL79* and *OsFBDUF54*. Furthermore, *OsFBDUF54* expression was higher in the sensitive variety KY131 than in the alkali-tolerant variety XBJZ and the opposite expression pattern were observed for *OsFBDUF53* and *LTPL79* (Fig. 5). We further found that *OsFBDUF54* of KY131 contained a 1-bp insertion in the CDS region relative to the sequence of XBJZ. So we assessed the response of *OsFBDUF54* mutant to alkali stress and found the mutant was more sensitive than wild-type plants to alkali stress (Fig. 6c), this may explain the observed variation in alkali tolerance. *MAF1* and *OsFBDUF54* belong to the same gene family. According to previous analyses, the overexpression of *MAF1* reduces abiotic stress tolerance and promotes root growth in rice (Yan et al. 2011). In the present study, *OsFBDUF54* may be a regulator of alkali tolerance at the bud stage in rice, but additional data are needed to verify this conclusion. The overexpression and RNA interference of *OsFBDUF54*, *OsFBDUF53* and *LTPL79* will further clarify the role of the candidate genes in the alkali stress signaling cascade and will be a focus of future studies.

## Conclusions

In the present study, RIL and natural populations were collected to evaluate the tolerance of alkalinity stress at the bud stage. A major QTL named *qAT11* on chromosome 11 was detected by linkage mapping. Chr11_21,999,659 located in *qAT11* was significantly associated with alkali tolerance on chromosome 11, as determined by GWAS. According to an LD analysis of the whole genome, a 218-kb region was selected for further study. Based on haplotype analysis and qRT-PCR, *LOC_Os11g37300*, *LOC_Os11g37320* and *LOC_Os11g37390* were the candidate genes contributing to alkaline tolerance in *japonica* rice. This study provides resources for breeding aimed at improving rice responses to alkalinity stress.

## Methods

### Plant Material

The linkage mapping population consisting of 184 RILs was derived from a cross between the *japonica* rice variety Kongyu131 (alkali-sensitive) and the upland rice variety Xiaobaijingzi (alkali-tolerant). The natural population for the GWAS comprised 295 *japonica* rice varieties, which were collected from the Liaoning, Jilin and Heilongjiang Provinces in China and other countries including the Republic of Korea, Japan, Russia and the Democratic People’s Republic of Korea. All 295 *japonica* rice varieties belong to temperate *japonica* rice. All of the varieties in this population were selected from a previous study (Li et al. 2019).

### Evaluation of Alkali Tolerance at the Bud Stage

Rice seeds were air-dried naturally, and kept at 55 °C for 5 days to break dormancy. They were then surface-sterilized with 1% sodium hypochlorite solution for 10 min, rinsed with sterile deionized water, and soaked in distilled water at 30 °C in dark conditions for 3 days. When the seed bud length was greater than or equal to half the length of the seed, 50 uniform germinated seeds were divided into two portions. Each was seeded in a well of a thin polypropylene plate with a nylon mesh bottom, with a single seed sown per well. Finally, the two portions of germinated seeds were separately transferred to distilled water and a culture solution containing 40 mM NaHCO_3_. The seeds were placed in an incubator with a 28/21 °C day/night cycle, natural sunlight, and 70% RH. The solution was changed every 2 days. After 10 days of alkaline stress, 10 seedlings with consistent growth were selected to measure the longest RL of the control and ARL. RRL was estimated to evaluate alkali tolerance. All phenotypes were tested in three replicates.

### QTL Mapping for Alkali Tolerance

A genetic linkage map was constructed from 184 RILs using 527 bin markers obtained from 10 K Array genotyping by target sequencing (GBTS) by MOLBREEDING Biotechnology Company (Shijiazhuang, China). The constructed linkage map covered 1874.85 cM of the rice genome with an average distance of 3.56 cM between markers (Fig. S1). The QTL analysis was based on the arithmetic mean values from three replicates for each trait by the inclusive composite interval mapping (ICIM) method implemented in QTL IciMapping Ver.4.2 (http://www.isbreeding.net). The LOD score threshold for QTL identification was 2.5 for each trait, and the walking speed was 1 cM.

### GWAS for Alkali Tolerance

In total, 788,369 SNPs with a minor allele frequency (MAF) of ≥5% and a missing rate of ≤20% were used to genotype 295 *japonica* rice accessions for GWAS. These were obtained by a preliminary study in our laboratory and the LD decay distance was 109 kb in the 295 *japonica* rice (Li et al. 2019). GWAS was performed using the mixed linear model (MLM) implemented in TASSEL 5.0 (Bradbury et al. 2007). The threshold for the identification of SNPs significantly associated with traits was set to *P* < 5.46 × 10^− 6^, determined by genetic type 1 error calculator (GEC; http://statgenpro.psychiatry.hku.hk/gec/), which calculates the effective number of independent markers. To determine significant sites, if two or more significant SNPs were located in the same LD interval, then these SNPs were treated as the same QTL, and the SNP with the smallest *P*-value was taken as lead SNP. The contribution rate of this SNP was the contribution rate of the QTL. Manhattan and Q–Q plots were created using the R package ‘qqman’ from the GWAS results.

### Haplotype Analysis of Candidate Gene

According to the LD decay analysis of the whole genome (LD decay = 109 kb), haplotype analysis was performed on all genes in the 218 kb region. The non-synonymous mutation SNPs in the exon region of all genes in the interval were extracted from 3KRGP’s RiceSNP-SeekDatabase website (https://snp-seek.irri.org/). These SNPs were used to perform haplotype analysis of all genes in the interval using DnaSP software. For genes with no significant difference in RRL between different haplotypes (≥ 10 materials), haplotype analysis was performed using SNPs in the promoter region (1.5 kb before ATG).

### RNA Extraction and Quantitative Real-Time PCR Analysis

After 72 h of alkalinity stress by 40 mM NaHCO_3_, rice roots from KY131 and XBJZ were sampled under alkaline and normal conditions. Total RNA was extracted from rice roots using the TranZol Up RNA Kit (TransGen Biotech, Beijing, China). Complementary DNA was synthesized from total RNA using the HiFiScript cDNA Synthesis Kit (Cwbio, Beijing, China). Quantitative real-time PCR (qRT-PCR) was performed using a Roche Light Cycler 2.10 system using 2× Fast qPCR Master Mix. Three biological replicates and three technical replicates were set for each sample. The mRNA levels of genes were determined relative to levels of the housekeeping gene *Actin1* (Li et al. 2019) as an internal control. Relative gene expression levels were determined using the 2^−ΔΔCt^ method (Livak and Schmittgen 2000). Data shown in figures and tables are mean values of three replicates.

### Candidate Gene Prediction, Sequencing, and Sequence Alignment

Combining the linkage mapping and GWAS results, based on haplotype analysis results, six genes were selected as candidates. Then, the corresponding candidate genes were cloned by PCR and sequenced gene and promoter region in KY131 and XBJZ. The sequence alignment was generated using DNAMAN.

### *OsFBDUF54* Mutant Plants

The homozygous T_1_ generation mutant seeds with Nipponbare background were obtained from BIOGLE GENETECH company (http://www.biogle.cn/) by CRISPR/Cas9 method in October 2019. The T_1_ generation seeds were planted in Hainan of China, and the homozygous T_2_ generation seeds (named NPB-mutant) were obtained in March 2020 and they were used for alkali tolerance identification.

## Supplementary information

**Additional file 1 : Fig. S1** Genetic linkage map and QTL mapping results.

**Additional file 2 : Table S1**. Descriptive statistics for root length under 0 mM NaHCO_3_ (C), and 40 NaHCO_3_ (A) Summary of the trait means in the parents, 184 recombinant inbred lines (RILs), and 295 rice accessions.

**Additional file 3 : Table S2** Summary of functional annotation results for genes in the candidate region on chromosome 11.

**Additional file 4 : Table S3** Primers used for qRT-PCR and relative expression in this study.

## Data Availability

The raw Illumina sequencing data from this study have been submitted to NCBI Sequence Read Archive (SRA) under the accession number PRJNA512109.

## Abbreviations

GWAS: Genome-wide association study
RL: Root length
ARL: Root length under alkali stress
RRL: Relative root length
MAF: Minor allele frequency
QTL: Quantitative trait loci
SNP: Single-nucleotide polymorphism
LD: Linkage disequilibrium
RIL: Recombinant inbred line
KY131: Kongyu131
XBJZ: Xiaobaijingzi
