# Supplementary figures and images for: QTL Mapping and Candidate Gene Analysis for Alkali Tolerance in Japonica Rice at the bud Stage Based on Linkage Mapping and Genome-Wide Association Study
Source: Rice (N Y). 2020 Jul 16;13:48. doi: 10.1186/s12284-020-00412-5 (PMC7364718; doi:10.1186/s12284-020-00412-5)

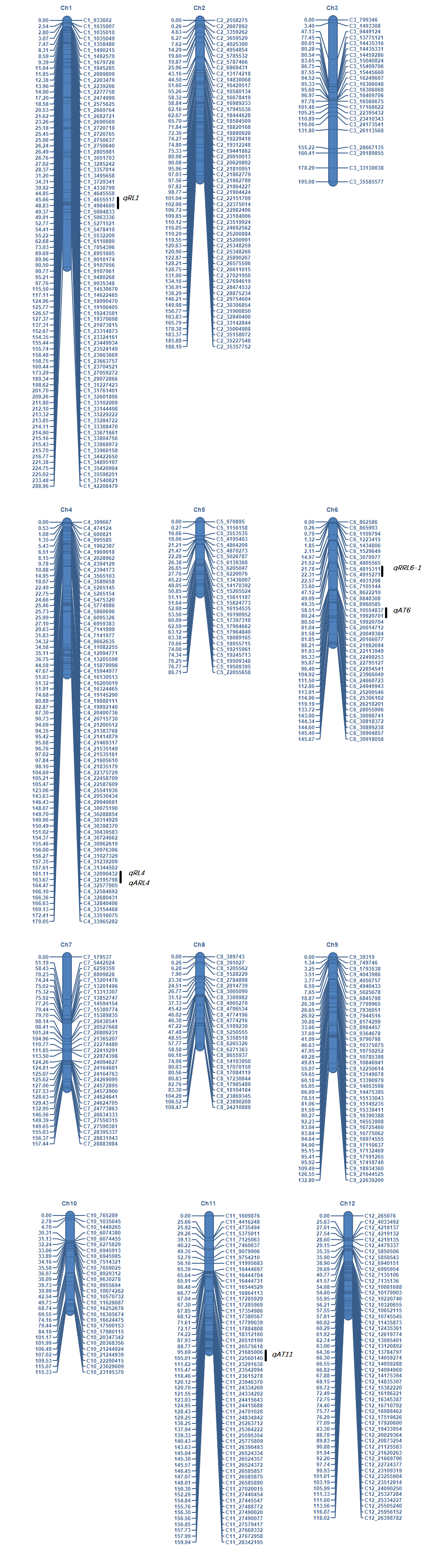

Supplement: Supplementary file 1 — Additional file 1 : Fig. S1 Genetic linkage map and QTL mapping results. [file 12284_2020_412_MOESM1_ESM.png]
